# Association between physical activity and frailty transitions in middle-aged and older adults: a nationwide longitudinal study

**DOI:** 10.1186/s12966-025-01725-8

**Published:** 2025-03-10

**Authors:** Li Huang, Zhenzhen Liang, Huajian Chen

**Affiliations:** 1 Faculty of Medical, Heidelberg University, Heidelberg, Germany; 2 Department of Epidemiology and Health Statistics, School of Public Health, Xinxiang Medical University, Xinxiang, China; 3 School of Public Health, Wenzhou Medical University, Wenzhou, China

**Keywords:** Middle-aged and older adult, Physical activity, Frailty, Frailty transitions

## Abstract

**Background:**

Frailty has become an important health problem in the middle-aged and older people population. Physical activity (PA) is a key intervention for frailty prevention and management. However, studies of the association between COVID-19 pre-pandemic PA and the worsening or improvement of frailty during the pandemic remain unclear.

**Methods:**

This longitudinal cohort study used data from the English Longitudinal Study of Ageing (ELSA), including participants aged 50 and over. Three intensities of PA (vigorous, moderate, and mild) were categorized as less than once per week and at least once per week, respectively, based on participant self-report. The frailty index (FI) assessed the frailty status, defining frailty as FI ≥ 25. Logistic regression was applied to examine the association between PA and frailty, estimating odds ratios (OR) and 95% confidence intervals (95% CI).

**Results:**

Of the 4379 non-frail participants at baseline (median age 67, 54.9% female), 8.1% developed frailty during a mean follow-up of 3.5 years. Among 564 frail participants at baseline (median age 71, 66.5% female), 17.9% regained health. Compared to those engaging in PA less than once per week, participants who engaged in vigorous (OR: 0.47 [95% CI: 0.35–0.62]), moderate (OR: 0.37 [95% CI: 0.29–0.48]), or mild (OR: 0.38 [95% CI: 0.26–0.56]) PA at least once a week had a lower risk of frailty worsening. Additionally, participants who engaged in moderate (OR: 2.04 [95% CI: 1.29–3.21]) or mild (OR: 2.93 [95% CI: 1.54–5.58]) PA at least once a week had a higher likelihood of frailty improvement. Sensitivity analyses based on comprehensive PA levels confirmed these findings. Participants who maintained at least one PA per week had lower frailty worsening (Vigorous, OR: 0.20 [95%CI: 0.12–0.33]; Moderate, OR: 0.13 [95%CI: 0.09–0.19]; Mild, OR: 0.20 [95%CI: 0.11–0.38]) and higher frailty improvement rates (Moderate, OR: 3.43 [95%CI: 1.93–6.11]; Mild, OR: 4.65 [95%CI: 1.90-11.42]). In addition, individuals (Vigorous, OR: 0.35 [95%CI: 0.20–0.60]; Moderate, OR: 0.36 [95%CI: 0.22–0.56]) who transitioned from inactive to active also exhibited a lower risk of frailty.

**Conclusions:**

This study emphasized the critical role of PA in preventing and improving frailty in middle-aged and older people, especially during the COVID-19 pandemic. Our study also highlighted the importance of maintaining PA habits to reduce frailty risk and promote its improvement. Also, the study indicated that individuals who transitioned from inactive to active had a lower risk of frailty. These findings enriched the understanding of the association between PA and frailty and provided valuable insights for addressing the health impact of future pandemics on middle-aged and older people.

**Supplementary Information:**

The online version contains supplementary material available at 10.1186/s12966-025-01725-8.

## Background

Frailty is characterized by a decline in the function and reserve capacity of multiple physiological systems, leading to a weakening of the body’s defenses and making it more vulnerable to stressful events [1–3]. This phenomenon is increasingly becoming an emerging burden on global health [4]. The indirect effects of the COVID-19 pandemic, mainly due to restriction measures, have been extensively studied, revealing significant difficulties in accessing care for individuals with chronic diseases, as well as a marked increase in psychosocial disorders (such as depression, anxiety, and loneliness), malnutrition (both over- and under-nutrition), and cognitive impairments, all of which may contribute to the incidence and progression of frailty [5–11]. Frailty is strongly associated with a wide range of adverse health outcomes, including disability, dependence, falls, long-term care needs, and premature mortality [2, 4, 12, 13–15]. As the global population ages, the prevalence of frailty is gradually increasing, portending a future in which more and more middle-aged and older people will face frailty-related challenges [16]. However, recent evidences suggested that frailty is a dynamic process that can be slowed down or even reversed with effective interventions [1, 17, 18]. Frailty transitions are the processes by which individuals change between different frailty states, including transitions between non-frailty, pre-frailty, and frailty states [19]. Particularly in clinical care pathways, the frailty transition approach aids clinical decision-making by simplifying categorization, while an understanding of the frailty transition helps to identify the best population to target for intervention [19]. Therefore, to increase attention to frailty in this population and to enhance their quality of life, it is particularly important to identify key factors that prevent and reverse frailty.

Exercise plays a key role in preventing and reversing adult frailty [17]. Physical activity (PA) has been recognized as a potentially effective strategy for preventing or reversing frailty [20] by improving vascular function and enhancing antioxidant capacity, positively affecting immune function, skeletal muscle function, and neuromuscular control [21–23]. For adults, 150–300 min of moderate-intensity physical activity, 75–150 min of vigorous-intensity physical activity, or an equal combination of moderate- and vigorous-intensity aerobic physical activity per week is beneficial for maintaining good health [24]. Although higher levels of PA may be accompanied by a gradual waning of relative benefits, there is no doubt that more PA has a positive effect on achieving optimal health [24]. A meta-analysis also suggested that an increase in exercise would increase muscle capacity [25]. Therefore, individuals can effectively prevent frailty through PA [26]. A cohort study from China found that after 3.1 years of follow-up, consistent aerobic exercise reduced the risk of frailty by 26% [27]. Similarly, another cohort study from Brazil found that people with less than 150 min of PA per week accompanied by excessive sedentary time were more likely to experience symptoms of frailty [28]. In addition, a cohort study from the UK also showed that moderate and intense PA significantly slowed the progression of frailty in older people [29]. PA has also been shown to be remarkably effective in reversing states of frailty. A randomized controlled trial (RCT) of 163 frail adults aged 65 years and older has shown that a new mixed exercise intervention program has a significant effect on improving frailty. After a 24-week comprehensive exercise intervention, 41.7% of participants who were initially in a frail state successfully reversed their frailty status [30]. Similarly, an RCT-based meta-analysis reported that exercise interventions (e.g., tai chi) were more effective in improving physical performance in frailty patients compared to non-exercise populations [31]. In the long term, consistent PA will provide significant benefits to the individual. A European-based cohort study found that maintaining regular PA can help maintain or improve an individual’s overall, physical, psychological, and social frailties [32]. Another Singaporean cohort study also found that consistent mild PA (e.g., housework) was associated with a lower risk of developing frailty [33]. All of the above studies showed a strong association between PA and frailty.

Following the COVID-19 pandemic, there was a significant change in daily activity patterns globally, with a generalized decline in PA levels accompanied by an increase in the prevalence of frailty as a result of restrictive measures and concerns about public health safety [6, 11, 34]. However, there is still insufficient research on the impact of pre-COVID-19 pandemic PA status on frailty worsening or improvement during the pandemic. To fill this gap in association studies, we used data from the English Longitudinal Study of Aging (ELSA) to analyze in detail the association between different intensities of PA (e.g., mild, moderate, and vigorous PA) before the pandemic and frailty worsening or improvement during the pandemic. In addition, we further assessed the longitudinal association between consistent PA and the worsening or improvement of frailty in this population during a mean follow-up period of 3.5 years. This study not only helps to reveal the potential role of PA in preventing or reversing frailty but also provides a scientific basis for optimizing public health intervention strategies, especially in the context of the COVID-19 pandemic that has profoundly affected physical activity patterns and health outcomes.

## Methods

### Study design and population

The ELSA is the only ongoing longitudinal survey of the aging population in the UK [35–37]. The ELSA population is a representative sample of men and women aged 50 and over living in England. The study began in 2002 and questionnaires have been administered every two years since then. So far, a total of 10 waves have been collected by ELSA, with the latest available wave (Wave 10) collected from May 2021 to March 2023. To explore the association between pre-pandemic PA and the onset and recovery of frailty during the pandemic, we selected Wave 9, which was collected between July 2018 and August 2019, as a baseline. The multidisciplinary nature of ELSA allows us to explore the complex associations between geriatric health and quality of life, thus making these data potentially the most appropriate for our analysis. The ELSA study was approved by the National Research Ethics Service (London Multicenter Research Ethics Committee [MREC 01/2/91]) and all participants signed a written informed consent form. The study was conducted under all relevant ethical regulations.

**Fig. 1:**
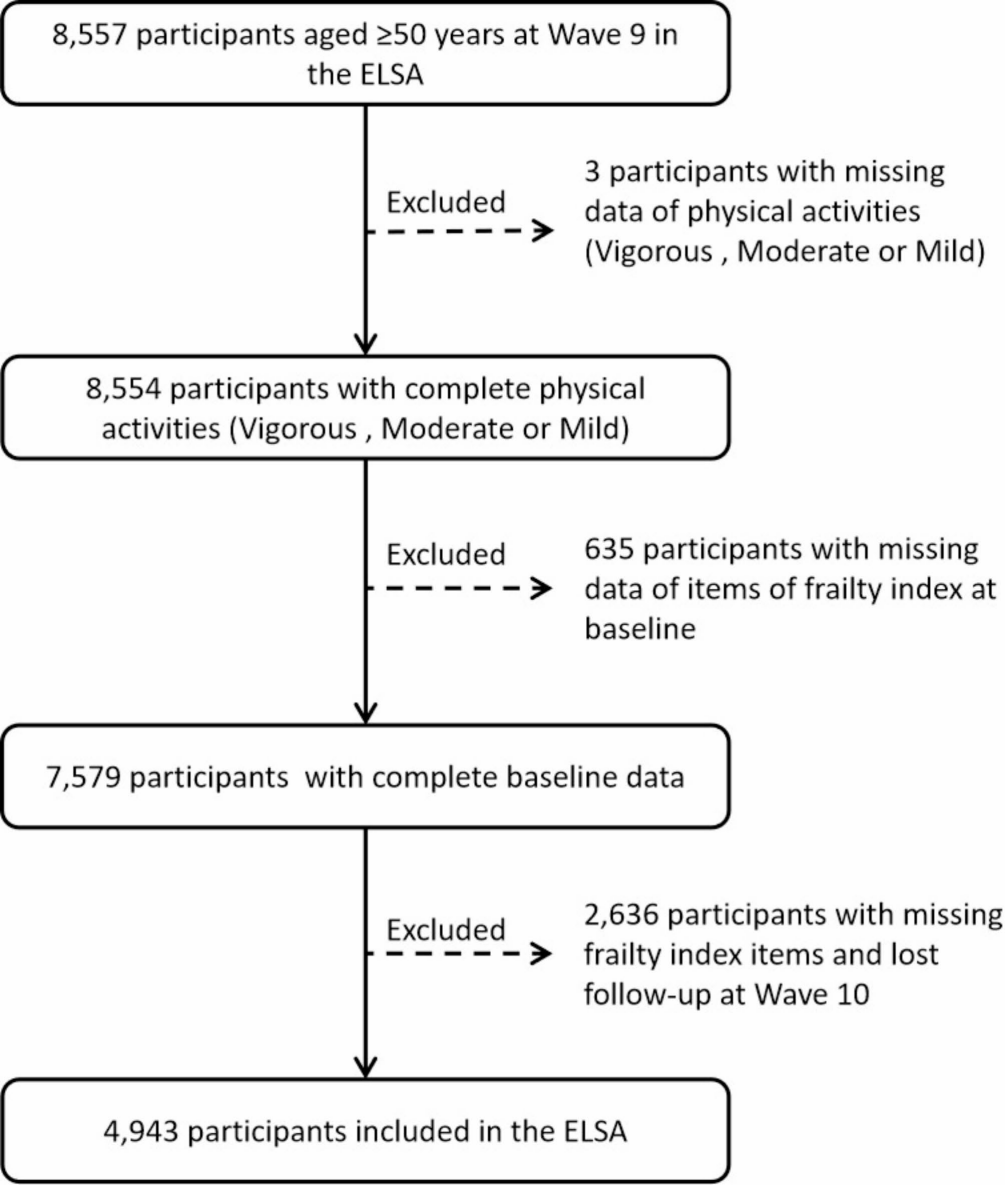
Selection process of the study population. ELSA, English Longitudinal Study of Ageing

Figure 1 shows the selection process for the study population. We recruited a total of 8557 participants at baseline (Wave 9) based on age ≥ 50 years. Participants were excluded if they did not provide complete PA information (including vigorous, moderate, or mild), or if the frailty index (FI) item was missing at baseline, or if the FI was not reassessed at the next wave (Wave 10) of follow-up (lost to follow-up), or if the FI item was missing at the next wave of follow-up (Wave 10). Based on the inclusion and exclusion criteria, a total of 4943 participants were included in this study, divided according to baseline FI thresholds, of which 4379 were non-frailty participants and 564 were frailty participants. Of the eligible participants, 4,377 non-frailty participants and 564 frailty participants could provide complete PA information (including vigorous, moderate, or mild) at the second resurvey (Wave 10). These 4941 participants were included in the analysis of the association between PA changes and frailty transition (worsening or improvement). Missing covariates were filled in by multivariate interpolation of chained equations using the mice package in R [38, 39].

### Assessment of frailty

Frailty was assessed by FI, which is calculated as the accumulation of age-related health deficits. We constructed the FI following the standard procedure described previously, which requires at least 30 deficits to construct the FI [40, 41]. Therefore, we selected 32 items to construct the FI, including comorbidities, physical functioning, disability, depression, and cognitive variables (Supplementary Table S1). Item 32 is a continuous variable ranging from 0 to 1, with higher values indicating lower cognitive function. For each participant, the 32-FI was calculated as the sum of current health deficits divided by 32 and multiplied by 100. Thus, the 32-FI is a continuous variable ranging from 0 to 100, with higher scores indicating greater frailty. Based on previous studies, we defined 32-FI ≥ 25 as frailty [1, 42].

### Physical activity

At baseline (Wave 9) and in the follow-up interview (Wave 10), PA was measured by asking participants to perform vigorous, moderate, and mild PA frequencies. Before the participants answered, the interviewer provided specific examples of different PAs to help them understand the various intensities of PA. Examples of mild activities included laundry and home repairs; moderate-intensity activity included gardening, cleaning the car, walking at moderate pace, dancing, and floor or stretching exercises; and vigorous intensity included running/jogging, swimming, cycling, aerobics/gym workout, tennis, and digging with a spade. There are four options for each question: rarely or never, one to three times a month, once a week, and many times a week [43]. Following the experience of previous studies, we categorized each PA into two groups based on their answers: less than once a week (rarely or never and one to three times a month) and at least once a week (once a week and many times a week) [44–48].

Based on the change in PA frequency between baseline and the second follow-up (mean interval of 3.5 years), we categorized the changes in vigorous, moderate, and mild PA into four groups, respectively: Consistently active (PA frequency of at least weekly for both surveys), inactive to active (baseline PA frequency of less than weekly and follow-up PA frequency of at least weekly), active to inactive (baseline PA frequency of at least weekly and follow-up PA frequency of less than weekly), and inactive (PA frequency of less than weekly for both surveys).

### Covariates

The covariates in this study included age, sex, ethnicity, education, marital status, smoking status, drinking status, use of hypertension medication, and use of diabetes medication. Ethnicity was categorized as white and non-white. Education level was divided into two categories as high school and above and below high school. Marital status was classified as married or partnered and other marital status (unmarried, separated, divorced, or widowed). Smoking status was divided into three categories as current smokers, former smokers, and never smokers. Drinking status was assessed based on the frequency of drinking in the last 12 months and was categorized as ≥ 1 time per month, < 1 time per month, and never drinkers. Assessment of medication use included whether the respondent had hypertension medication use and whether they had diabetes medication use (taking glucose-lowering medication and insulin injections).

The data on coronavirus symptoms comes from the ELSA COVID-19 Study, which is based on earlier investigations following the 2020 COVID-19 outbreak. Coronavirus symptoms include high temperature, a new continuous cough, shortness of breath or trouble breathing, fatigue, loss of sense of smell or taste, diarrhoea, abdominal pain, and loss of appetite. We summarized these symptoms into a total score ranging from 0 to 8, indicating the number of coronavirus symptoms experienced by each participant (Supplementary Table S2). This number of coronavirus symptoms was included as an additional covariate in the model and reanalyzed.

### Statistical analysis

Characteristics of study participants were presented as means (standard deviations [SD]) or medians (interquartile ranges [IQR]) for continuous measures and as percentages for categorical variables. The chi-square test was used for categorical variables, the t-test for normally distributed variables, and the rank sum test for non-normally distributed data.

During a mean follow-up period of 3.5 years, we used logistic regression to calculate odds ratios (ORs) and 95% confidence intervals (95% CIs) to examine the associations between the frequency of PAs (mild, moderate, vigorous, and any intensity) and the comprehensive level of PA and the frailty worsening or frailty improvement. To control for the effect of potential confounders on the study results, two models were constructed for analysis. These were: Model 1, unadjusted; Model 2, adjusted for age, sex, ethnicity, education level, marital status, smoking status, drinker status, use of high blood pressure medication, and use of diabetes medication. Using the same methodology, we analyzed the association of consistently active PAs with frailty worsening and improvement, using discontinuously active PAs as a reference. In subgroup analyses, we used fully adjusted models to explore whether the associations of PA (mild, moderate, vigorous, and any intensity) and comprehensive PA levels with frailty worsening or improvement remained stable across age groups (50–64 versus ≥ 65 years) and gender. In the sensitivity analysis, we constructed a comprehensive PA level based on the categorization in other ELSA studies and divided it into four groups [49, 50]: inactive, low (only mild activity at least once a week), moderate (at least moderate but no vigorous activity at least once a week) and high (any vigorous activity at least once a week).

Data management and statistical analysis were performed using R software (version 4.3.1). All statistical tests were two-sided, with *P* less than 0.05 considered statistically significant.

## Results

### Characteristics of the study participants

A total of 4943 eligible participants were included in the study, including 4379 who transitioned from no frailty to frailty (median [IQR] age: 67 [61, 72] years, 54.9% female) and 564 who transitioned from frailty to no frailty (median [IQR] age: 71 [64, 78] years, 66.5% female). As shown in Table 1, during a mean follow-up period of 3.5 years, the incidence of frailty was 8.1% among participants who were non-frail at baseline, while the rate of improvement in frailty was 17.9% among participants who were frail at baseline. In addition, participants with worsening frailty exhibited the following characteristics compared to those without frailty: older age, lower educational attainment, higher rates of being divorced, widowed, or unmarried, higher rates of being current or former smokers, less than 1 alcoholic drink per month, higher use of antihypertensive and diabetic medications, and lower rates of vigorous, moderate, or light physical activity per week. Participants with improved frailty were more likely to be moderately or mildly physically active at least once a week compared to those without improved frailty.

**Table 1:** Baseline characteristics of participants transitioning between frailty States (non-frailty to frailty and frailty to non-frailty)

Variables	Transition from non-frailty to frailty				Transition from frailty to non-frailty			
	Overall	Frailty ^a^	Non-frailty ^a^	*P* value	Overall	Frailty ^a^	Non-frailty ^a^	*P* value
Number (%)	4379	355 (8.1)	4024 (91.9)		564	463 (82.1)	101 (17.9)	
Age, median (IQR), years	67 (61, 72)	71 (65, 78)	66 (60, 72)	**< 0.001**	71 (64, 78)	71 (64, 78)	70 (64, 77)	0.472
Sex, n (%)				0.057				0.230
Male	1975 (45.1)	143 (40.3)	1832 (45.5)		189 (33.5)	150 (32.4)	39 (38.6)	
Female	2404 (54.9)	212 (59.7)	2192 (54.5)		375 (66.5)	313 (67.6)	62 (61.4)	
Ethnicity, n (%)				0.149				0.796
Non-White	151 (3.4)	17 (4.8)	134 (3.3)		26 (4.6)	21 (4.5)	5 (5)	
White	4228 (96.6)	338 (95.2)	3890 (96.7)		538 (95.4)	442 (95.5)	96 (95)	
Education level, n (%)				**< 0.001**				0.924
Below high school	630 (14.4)	91 (25.6)	539 (13.4)		182 (32.3)	149 (32.2)	33 (32.7)	
High school and above	3749 (85.6)	264 (74.4)	3485 (86.6)		382 (67.7)	314 (67.8)	68 (67.3)	
Marital status, n (%)				**< 0.001**				0.468
Married or partnered	3302 (75.4)	218 (61.4)	3084 (76.6)		328 (58.2)	266 (57.5)	62 (61.4)	
Other marital status	1077 (24.6)	137 (38.6)	940 (23.4)		236 (41.8)	197 (42.5)	39 (38.6)	
Smoking status, n (%)				**0.004**				0.073
Current smoker	333 (7.6)	35 (9.9)	298 (7.4)		58 (10.3)	52 (11.2)	6 (5.9)	
Ever smoker	2177 (49.7)	197 (55.5)	1980 (49.2)		324 (57.4)	270 (58.3)	54 (53.5)	
Never smoker	1869 (42.7)	123 (34.6)	1746 (43.4)		182 (32.3)	141 (30.5)	41 (40.6)	
Drinker status, n (%)				**< 0.001**				0.578
< 1 per month	572 (13.1)	55 (15.5)	517 (12.8)		124 (22)	100 (21.6)	24 (23.8)	
>= 1 per month	3387 (77.3)	244 (68.7)	3143 (78.1)		306 (54.3)	249 (53.8)	57 (56.4)	
Never drinker	420 (9.6)	56 (15.8)	364 (9)		134 (23.8)	114 (24.6)	20 (19.8)	
Take HBP medication, n (%)				**< 0.001**				**0.048**
No	3071 (70.1)	191 (53.8)	2880 (71.6)		246 (43.6)	193 (41.7)	53 (52.5)	
Yes	1308 (29.9)	164 (46.2)	1144 (28.4)		318 (56.4)	270 (58.3)	48 (47.5)	
Take diabetes medication, n (%)				**< 0.001**				0.100
No	4062 (92.8)	292 (82.3)	3770 (93.7)		440 (78)	355 (76.7)	85 (84.2)	
Yes	317 (7.2)	63 (17.7)	254 (6.3)		124 (22)	108 (23.3)	16 (15.8)	
Vigorous PA, n (%)				**< 0.001**				0.485
< 1 per week	2622 (59.9)	286 (80.6)	2336 (58.1)		528 (93.6)	435 (94)	93 (92.1)	
≥ 1 per week	1757 (40.1)	69 (19.4)	1688 (41.9)		36 (6.4)	28 (6)	8 (7.9)	
Moderate PA, n (%)				**< 0.001**				**0.001**
< 1 per week	556 (12.7)	110 (31)	446 (11.1)		363 (64.4)	312 (67.4)	51 (50.5)	
≥ 1 per week	3823 (87.3)	245 (69)	3578 (88.9)		201 (35.6)	151 (32.6)	50 (49.5)	
Mild PA, n (%)				**< 0.001**				**0.002**
< 1 per week	232 (5.3)	44 (12.4)	188 (4.7)		139 (24.6)	126 (27.2)	13 (12.9)	
≥ 1 per week	4147 (94.7)	311 (87.6)	3836 (95.3)		425 (75.4)	337 (72.8)	88 (87.1)	
PA (Any), n (%)				**< 0.001**				**0.007**
< 1 per week	124 (2.8)	32 (9)	92 (2.3)		131 (23.2)	118 (25.5)	13 (12.9)	
≥ 1 per week	4255 (97.2)	323 (91)	3932 (97.7)		433 (76.8)	345 (74.5)	88 (87.1)	
PA levels				**< 0.001**				**0.007**
Inactive	124 (2.8)	32 (9)	92 (2.3)		131 (23.2)	118 (25.5)	13 (12.9)	
Low	390 (8.9)	75 (21.1)	315 (7.8)		219 (38.8)	183 (39.5)	36 (35.6)	
Moderate	2108 (48.1)	179 (50.4)	1929 (47.9)		178 (31.6)	134 (28.9)	44 (43.6)	
High	1757 (40.1)	69 (19.4)	1688 (41.9)		36 (6.4)	28 (6)	8 (7.9)	

^a^ The frailty status of the participants in wave 10

HBP, high blood pressure; PA, Physical activity

### Association between PA frequency and the frailty worsening

The associations of vigorous, moderate, mild, and any intensity PA frequency with the frailty worsening during a mean follow-up period of 3.5 years are shown in Table 2. Among participants who were not frail at baseline, individuals who engaged in any PA at least once per week had a 68.0% (95% CI: 50.0 − 80.0%) lower risk of frailty worsening compared to those whose frequency was less than once per week. Compared to participants who attended less than once a week, vigorous, moderate and mild PA at least once a week were all significantly associated with lower frailty worsening, with moderate PA showing the strongest negative association (OR = 0.37, 95% CI: 0.29–0.48), followed by mild PA (OR = 0.38, 95% CI: 0.26–0.56) and vigorous PA (OR = 0.47, 95% CI: 0.35–0.62).

**Table 2:** Association between PA frequency and frailty worsening

Variables	Model 1		Model 2	
	OR (95% CI)	*P*	OR (95% CI)	*P*
**Vigorous PA**				
<1 per week	1 (Reference)		1 (Reference)	
≥ 1 per week	0.33 (0.25–0.44)	**< 0.001**	0.47 (0.35–0.62)	**< 0.001**
**Moderate PA**				
< 1 per week	1 (Reference)		1 (Reference)	
≥ 1 per week	0.28 (0.22–0.35)	**< 0.001**	0.37 (0.29–0.48)	**< 0.001**
**Mild PA**				
< 1 per week	1 (Reference)		1 (Reference)	
≥ 1 per week	0.35 (0.24–0.49)	**< 0.001**	0.38 (0.26–0.56)	**< 0.001**
**PA (Any)**				
< 1 per week	1 (Reference)		1 (Reference)	
≥ 1 per week	0.24 (0.16–0.36)	**< 0.001**	0.32 (0.20–0.50)	**< 0.001**

Model 1: Unadjusted

Model 2: Adjusted for age, sex, ethnicity, education level, marital status, smoking status, drinker status, use of high blood pressure medication, and use of diabetes medication

PA, Physical activity

### Association between PAs frequency and the frailty improvement

Table 3 shows the association between the frequency of vigorous, moderate, mild, and any PA and frailty improvement over a mean follow-up of 3.5 years. Individuals who engaged in any PA at least once per week had a 160% (95% CI: 36 − 396%) increased odds of frailty improvement compared to participants who engaged in any PA less than once per week. Except for vigorous PA (OR = 1.24, 95% CI: 0.54–2.88), both moderate and mild PA, which was engaged in at least once a week, were significantly associated with higher rates of frailty improvement compared to individuals who engaged in PA less than once a week. Of these, mild PA showed the highest rate of frailty improvement (OR = 2.93, 95% CI: 1.54–5.58), followed by moderate PA (OR = 2.04, 95% CI: 1.29–3.21).

**Table 3:** Association between PA frequency and frailty improvement

Variables	Model 1		Model 2	
	OR (95% CI)	*P*	OR (95% CI)	*P*
**Vigorous PA**				
<1 per week	1 (Reference)		1 (Reference)	
≥ 1 per week	1.34 (0.59–3.03)	0.487	1.24 (0.54–2.88)	0.613
**Moderate PA**				
< 1 per week	1 (Reference)		1 (Reference)	
≥ 1 per week	2.03 (1.31–3.13)	**0.002**	2.04 (1.29–3.21)	**0.002**
**Mild PA**				
< 1 per week	1 (Reference)		1 (Reference)	
≥ 1 per week	2.53 (1.37–4.69)	**0.003**	2.93 (1.54–5.58)	**0.001**
**PA (Any)**				
< 1 per week	1 (Reference)		1 (Reference)	
≥ 1 per week	2.32 (1.25–4.30)	**0.008**	2.60 (1.36–4.96)	**0.004**

Model 1: Unadjusted

Model 2: Adjusted for age, sex, ethnicity, education level, marital status, smoking status, drinker status, use of high blood pressure medication, and use of diabetes medication

PA, Physical activity

### Subgroup analysis

To further explore the stability of the association between PA and frailty transition across population characteristics, including age and sex, subgroup analyses were conducted in this study (Table 4). Results showed that individuals who engaged in vigorous, moderate, mild, or any PA at least once per week had a significantly lower risk of frailty worsening than individuals who engaged in PA less than once per week. The associations remained stable across age groups (50–64 and ≥ 65 years) and sex (male and female) (All *P* for interaction > 0.05). Regarding frailty improvement, individuals who engaged in moderate, mild, or any PA at least once a week were associated with higher frailty improvement compared to participants who engaged in it less than once a week, and this remained stable across age groups (50–64 and ≥ 65 years) and gender (male and female) (All *P* for interaction > 0.05).

**Table 4:** Subgroup analysis

Variables	Transition from non-frailty to frailty		Transition from frailty to non-frailty	
	OR (95% CI)	*P* for interaction	OR (95% CI)	*P* for interaction
**Vigorous PA**				
**Age**		0.682		0.545
50–64	0.53 (0.31–0.87)		0.67 (0.03–5.05)	
>=65	0.41 (0.29–0.57)		1.38 (0.52–3.33)	
**Sex**		0.670		0.697
Male	0.47 (0.31–0.70)		1.26 (0.25–4.90)	
Female	0.41 (0.27–0.59)		1.28 (0.39–3.58)	
**Moderate PA**				
**Age**		0.864		0.833
50–64	0.37 (0.22–0.65)		1.92 (0.76–4.90)	
>=65	0.34 (0.25–0.46)		2.09 (1.23–3.56)	
**Sex**		0.894		0.392
Male	0.35 (0.23–0.54)		1.71 (0.77–3.75)	
Female	0.35 (0.25–0.49)		2.16 (1.21–3.90)	
**Mild PA**				
**Age**		0.871		0.186
50–64	0.32 (0.16–0.73)		1.68 (0.58–5.46)	
>=65	0.39 (0.25–0.60)		3.81 (1.75–9.58)	
**Sex**		0.401		0.784
Male	0.34 (0.21–0.55)		2.69 (1.14–7.05)	
Female	0.47 (0.26–0.88)		2.84 (1.16–8.60)	
**PA (Any)**				
**Age**		0.917		0.143
50–64	0.26 (0.11–0.72)		1.34 (0.46–4.35)	
>=65	0.31 (0.19–0.53)		3.51 (1.61–8.85)	
**Sex**		0.190		0.643
Male	0.23 (0.13–0.43)		2.13 (0.89–5.62)	
Female	0.42 (0.22–0.87)		2.72 (1.10–8.25)	

All analyses were adjusted for age, sex, ethnicity, education level, marital status, smoking status, drinker status, use of high blood pressure medication, and use of diabetes medication

PA, Physical activity

### Sensitivity analysis

Supplementary Table S3-5 shows the results of the sensitivity analyses. In general agreement with the main analyses, similar results were found in these sensitivity analyses. Results showed that participants with moderate levels (OR = 0.32, 95% CI: 0.20–0.51) and high levels (OR = 0.19, 95% CI: 0.11–0.31) of PA had a significantly lower risk of frailty worsening compared to inactive participants, and although the effect of low levels of PA did not reach statistical significance, its estimate of 0.70 has a tendency to move toward lower frailty worsening risk (Supplementary Table S3). In terms of frailty improvement, participants with low (OR = 2.06, 95% CI: 1.02–4.15) and moderate levels (OR = 3.30, 95% CI: 1.65–6.63) of PA had a higher rate of frailty improvement compared to inactive participants (Supplementary Table S4). In addition, as shown in Supplementary Table S5, the association of PA levels with worsening frailty was consistent across age and sex (All *P* for interaction > 0.05). Similarly, the association between PA levels and frailty improvement was consistent across age and gender (All *P* for interaction > 0.05). Subsequently, in analyzing the association between PA levels and worsening or improvement in frailty, we included coronavirus symptoms as a covariate in the model, and the results were consistent with previous analyses (Supplementary Tables S6-S8).

In analyzing the association between vigorous, moderate, and mild PA and worsening or improvement in frailty, we further included coronavirus symptoms as covariates in the model and reanalyzed them (Supplementary Tables S9-S10). Consistent with the results of the main analysis, these sensitivity analyses found similar results. In the association between PA and frailty worsening, results showed that participants with vigorous, moderate, and mild PA frequency of at least once a week had a lower risk of frailty worsening (Supplementary Table S9). In the association between PA and frailty improvement, results showed that participants with moderate and mild PA frequency of at least once a week had better frailty improvement (Supplementary Table S10).

### Association between consistent PAs and frailty

In the Wave 10 survey, among participants without frailty at baseline, 1,000 participants (22.8%) had consistently active vigorous PA, 3,265 participants (74.6%) had consistently active moderate PA, and 3,765 participants (86.0%) had consistently active mild PA. In terms of vigorous PA, compared to inactive participants, inactive to active (OR = 0.35, 95% CI: 0.20–0.60), active to inactive (OR = 0.65, 95% CI: 0.47–0.89), and continuously active (OR = 0.20, 95% CI: 0.12–0.33) participants had a lower risk of frailty worsening (Table 5). For moderate PA, participants who were inactive to active (OR = 0.36, 95% CI: 0.22–0.56), active to inactive (OR = 0.64, 95% CI: 0.44–0.92), and persistently active (OR = 0.13, 95% CI: 0.09–0.19) similarly showed demonstrated a lower risk of frailty worsening. However, in mild PA, only consistently active participants had a lower risk of frailty worsening compared to inactive participants (OR = 0.20, 95% CI: 0.11–0.38).

**Table 5:** Association between PA changes and frailty worsening

Variables	*n*	Model 1		Model 2	
		OR (95% CI)	*P*	OR (95% CI)	*P*
**Vigorous PA**					
Inactive	2202	1 (Reference)		1 (Reference)	
Inactive to Active	418	0.27 (0.16–0.45)	**< 0.001**	0.35 (0.20–0.60)	**< 0.001**
Active to Inactive	757	0.53 (0.39–0.72)	**< 0.001**	0.65 (0.47–0.89)	**0.008**
Consistently active	1000	0.12 (0.08–0.20)	**< 0.001**	0.20 (0.12–0.33)	**< 0.001**
**Moderate PA**					
Inactive	231	1 (Reference)		1 (Reference)	
Inactive to Active	324	0.29 (0.19–0.45)	**< 0.001**	0.36 (0.22–0.56)	**< 0.001**
Active to Inactive	557	0.57 (0.41–0.81)	**0.002**	0.64 (0.44–0.92)	**0.016**
Consistently active	3265	0.09 (0.07–0.13)	**< 0.001**	0.13 (0.09–0.19)	**< 0.001**
**Mild PA**					
Inactive	58	1 (Reference)		1 (Reference)	
Inactive to Active	174	0.44 (0.22–0.89)	**0.022**	0.53 (0.25–1.12)	0.095
Active to Inactive	380	0.59 (0.32–1.10)	0.098	0.61 (0.32–1.19)	0.148
Consistently active	3765	0.16 (0.09–0.29)	**< 0.001**	0.20 (0.11–0.38)	**< 0.001**

Model 1: Unadjusted

Model 2: Adjusted for age, sex, ethnicity, education level, marital status, smoking status, drinker status, use of high blood pressure medication, and use of diabetes medication

PA, Physical activity

Of the participants with frailty at baseline, 112 participants (19.9%) had consistently active moderate PA, and 312 participants (55.3%) had consistently active mild PA. Participants with consistently mild (OR = 4.65 95% CI: 1.90–11.42) or moderate (OR = 3.43, 95% CI: 1.93–6.11) PA showed a higher frailty improvement compared to inactive participants (Table 6).

**Table 6:** Association between PA changes and frailty improvement

Variables	*n*	Model 1		Model 2	
		OR (95% CI)	*P*	OR (95% CI)	*P*
**Moderate PA**					
Inactive	277	1 (Reference)		1 (Reference)	
Inactive to Active	86	1.96 (1.04–3.69)	**0.038**	1.86 (0.96–3.59)	0.067
Active to Inactive	89	1.50 (0.77–2.91)	0.232	1.51 (0.77–2.98)	0.234
Consistently active	112	3.36 (1.96–5.77)	**< 0.001**	3.43 (1.93–6.11)	**< 0.001**
**Mild PA**					
Inactive	92	1 (Reference)		1 (Reference)	
Inactive to Active	47	2.51 (0.79–7.95)	0.118	2.35 (0.72–7.64)	0.157
Active to Inactive	113	2.72 (1.03–7.16)	**0.043**	3.19 (1.18–8.64)	**0.022**
Consistently active	312	4.15 (1.74–9.89)	**0.001**	4.65 (1.90-11.42)	**< 0.001**

Model 1: Unadjusted

Model 2: Adjusted for age, sex, ethnicity, education level, marital status, smoking status, drinker status, use of high blood pressure medication, and use of diabetes medication

PA, Physical activity

Subsequently, we also included coronavirus symptoms as covariates for reanalysis (Supplementary Tables S11-S12). We found the results of the reanalysis to be consistent with the results of the main analysis. In the association between PA changes and frailty worsening, results showed that participants who were consistently active (vigorous, moderate, and mild PA), inactive to active (vigorous and moderate PA), and active to inactive (vigorous PA) were significantly associated with a lower risk of frailty worsening compared to those who were inactive (Supplementary Table S11). In the association between PA change and frailty improvement, results showed that participants who were consistently active (moderate and mild PA) had better rates of frailty improvement compared to those who were inactive (Supplementary Table S12).

## Discussion

The main finding of this study highlights the important role of PA in frailty transitions, particularly in the reciprocal transitions between non-frail and frail states. This study, based on a nationally representative sample of middle-aged and older people, found that engaging in vigorous, moderate, and mild PA at least once per week was associated with a significantly lower frailty worsening compared with individuals who engaged in PA less than once per week. It was also observed that in addition to vigorous PA, moderate and mild PA with participation at least once a week was significantly associated with higher rates of frailty improvement. Subgroup analyses showed that the association between the frequency of PA of different intensities and the worsening or improvement of frailty was consistent across age groups and sex. The results of the sensitivity analysis were generally consistent with the main analyses. In addition, we further demonstrated that consistent PA engagement was not only associated with a lower frailty worsening, but also contributed to frailty improvement in frail individuals over an average of 3.5 years of follow-up. Our study also found that among participants who transitioned from inactive to active, moderate and vigorous PA, except for mild PA, were significantly associated with a lower risk of frailty worsening. These findings provide important support for healthcare planning and provide evidence for the use of PA in the prevention and management of frailty in middle-aged and older people. The study of the association between pre-pandemic PA levels and the worsening or improvement of frailty during a pandemic can help to identify high-risk groups and inform the development of effective interventions to reduce the health burden of future pandemics concerning frailty.

Previous studies have demonstrated a negative correlation between PA and risk of frailty. A longitudinal study from Singapore found that mild PA (e.g., housework) was significantly associated with a lower incidence of frailty in older people [33]. Another longitudinal cohort study from the UK found that mild PA was associated with a reduced risk of type 2 diabetes in older people [48]. Our findings similarly suggested that mild PA was effective in reducing the incidence of frailty in middle-aged and older people, further emphasizing the importance of mild PA in the management of frailty in middle-aged and older people. A cross-sectional study of 638 older people aged 70 years or older showed that longer PA engagement, less sedentary time, and more frequent PA were significantly associated with a lower prevalence of frailty [51]. Additionally, a study involving 511 older people in the community also found that healthy peers engaged in moderate to vigorous PA for longer periods compared to frail or pre-frailty individuals [52]. Our findings also suggest that the frequency of PA of different intensities (mild, moderate, and vigorous) significantly reduces the incidence of frailty when performed at least once a week. In terms of comprehensive PA levels, participants at moderate to high levels of PA demonstrated a lower risk of frailty worsening compared to inactive individuals. Additionally, our study found that consistent participation in PA, regardless of intensity (whether mild, moderate, or vigorous) significantly reduces the risk of frailty worsening in middle-aged and older adults. This suggests that, prior to the pandemic, more frequent engagement in PA at various intensities can effectively prevent the frailty worsening, while maintaining regular PA habits further helps to reduce frailty worsening. We found that participants who transitioned from inactive to active had a lower risk of frailty worsening, suggesting that even transitioning from an inactive state to an active one can significantly reduce the risk of frailty worsening. This result further supports the crucial role of PA in frailty prevention and highlights the long-term benefits of early intervention through PA to reduce frailty risk.

The dynamic nature of frailty means that intervention at the right time has the potential to reverse this state [53, 54]. A study from China showed that maintaining a healthy sleep pattern during an 8-year median follow-up period was significantly associated with an increased likelihood of frailty improvement [55]. Another UK longitudinal cohort study found a significant association between increased vitamin D levels and the transition from frailty to health [56]. However, studies on frailty improvement are still relatively rare, especially for middle-aged and older people, and the role of PA in frailty improvement has not been fully explored. In our study, we found that middle-aged and older people who performed moderate or mild PA at least once a week were more likely to improve frailty. In addition, middle-aged and older people with low to moderate levels of comprehensive PA showed higher rates of frailty improvement. Regular moderate-intensity PA improves all aspects of human health and is widely recognized as an effective strategy for preventing and treating many diseases [57]. A study of older people found that low-intensity activity increased deep sleep and improved memory functioning [58]. Another study of dialysis patients showed that a personalized low-intensity home exercise program not only improved their physical performance but also positively impacted their cognitive functioning and the quality of their social interactions [59]. Despite the controversy regarding the effects of low-intensity PA on physical health, our study further confirmed that low-intensity PA plays an important role in the frailty worsening and frailty improvement. In addition, our study found that consistent performance of low- and moderate-intensity PA had equally significant positive effects on improving frailty in a population of middle-aged and older people.

Although our study found that the OR between vigorous PA and frailty improvement was 1.23 and did not reach statistical significance and that from a physiological point of view, resistance exercises of low to moderate intensity, as well as a moderate amount of resistance exercise, have a clear advantage in terms of safety, efficacy, and acceptability in older frail patients [60]. However, this does not mean that vigorous PA lacks benefits for frailty groups. In fact, implementing targeted vigorous PA interventions for frailty will help them improve. An RCT involving 94 frail older people found that 10 weeks of high-intensity progressive resistance training significantly improved participants’ muscle strength and gait speed [61]. Another RCT study of 161 frailty older people also showed that 12 weeks of high-intensity resistance training was not only effective in improving muscle strength in older people but also significantly improved gait speed [60]. Therefore, we believe that moderate planning and implementation of high-intensity exercise interventions, while ensuring safety, should be considered as an important strategy to enhance the health status of frailty individuals in middle-aged and older adults.

The evidence for the health benefits of exercise for middle-aged and older people is well established. A meta-analysis based on a non-clinical adult population showed that even when running was performed only once per week, individuals who participated in running significantly reduced all-cause mortality, cardiovascular mortality, and cancer mortality compared to those who did not run [44]. Moreover, another meta-analysis further found that physically active older people had a significantly lower risk of all-cause mortality, cardiovascular disease, breast and prostate cancer, bone fractures, recurrent falls, impairment in activities of daily living, functional limitations, cognitive decline, dementia, Alzheimer’s disease, and depression [62]. It has been well evidenced that exercise promotes positive physiological adaptive responses by maintaining and restoring homeostasis of the organism’s internal environment at the organismal, tissue, cellular, and molecular levels [57]. These adaptive changes not only help to improve overall health but are also effective in preventing the onset of many pathologies. In addition, the amount of exercise is an important factor influencing intracellular processes of muscle anabolism and is also the most easily modified variable affecting the function of the major muscles, with important implications for muscle hypertrophy and health [63]. Even in cases where individuals are unable or unwilling to undergo relatively high-intensity resistance training, there are still significant health benefits associated with relatively high-volume training [60].

Our study has significant advantages. First, to our knowledge, this is the first analysis of the association between different intensities of PA before the COVID-19 pandemic and the worsening or improvement of frailty during the pandemic. This study provided an important basis for identifying high-risk groups and provided scientific support for developing effective interventions to reduce the health burden of frailty in future pandemics. Second, this study is based on nationally representative longitudinal cohort data, which allows for more accurate inferences of causality. Third, we also conducted a concurrent study on the association between the level of comprehensive PA and the worsening or improvement of frailty, which further enriched the research on the association between PA and frailty. Fourth, this study provided individuals with an empirical evidence base on the association between dynamic PA and frailty transition. We found that consistent participation in PA (at least once per week) significantly improved health regardless of intensity. In addition, we found that participants who transitioned from inactive to active also exhibited a lower risk of frailty worsening, further emphasizing the important role of physical activity in frailty prevention. Finally, we included the number of coronavirus symptoms from the follow-up survey as a covariate and re-conducted all analyses, with the results consistent with the main analysis.

This study also has some limitations. First, self-reported data inevitably suffer from recall bias. Second, in this study, we categorized frailty into two levels without further subdividing it into pre-frailty stages. Third, since PA of different intensities was only categorized into two levels, it was not possible to precisely calculate the amount of exercise suitable for middle-aged and older people. Therefore, we constructed a comprehensive PA level by combining PA of different intensities to further validate the association between PA and frailty. Finally, this study focused specifically on middle-aged and older people aged 50 and over in the UK. The findings may not apply to younger age groups or other races/ethnicities due to differences in the underlying cultural and genetic backgrounds of this particular population.

## Conclusion

In conclusion, this study suggested that performing PA of different intensities at least once a week was associated with a significant reduction in the risk of frailty worsening in middle-aged and older people. In addition, moderate or mild PA at least once a week was strongly associated with frailty improvement in middle-aged and older people. Our study further emphasized the importance of maintaining PA habits, especially during a pandemic, and that engaging in regular physical activity is an effective strategy that can significantly reduce the risk of frailty and promote health recovery. Also, the study indicates that individuals who transitioned from inactive to active had a lower risk of frailty. The findings not only enriched the understanding of the association between PA and frailty but also provided a valuable rationale for public health interventions, particularly in addressing the health impact of future pandemics on middle-aged and older people populations.

## Electronic supplementary material

Below is the link to the electronic supplementary material.

Supplementary Material 1

## Data Availability

The English Longitudinal Study of Ageing data were available from https://www.elsa-project.ac.uk/.

## Abbreviations

PA: Physical activity
FI: Frailty index
RCT: randomized controlled trial

## References

[1] Hoogendijk EO, Afilalo J, Ensrud KE, Kowal P, Onder G, Fried LP. Frailty: implications for clinical practice and public health. Lancet. 2019;394(10206):1365–75. 31609228 10.1016/S0140-6736(19)31786-6

[2] Clegg A, Young J, Iliffe S, Rikkert MO, Rockwood K. Frailty in elderly people. Lancet. 2013;381(9868):752–62. 23395245 10.1016/S0140-6736(12)62167-9 PMC4098658

[3] Collard RM, Boter H, Schoevers RA, Oude Voshaar RC. Prevalence of frailty in community-dwelling older persons: a systematic review. J Am Geriatr Soc. 2012;60(8):1487–92. 22881367 10.1111/j.1532-5415.2012.04054.x

[4] Dent E, Hanlon P, Sim M, Jylhävä J, Liu Z, Vetrano DL, Stolz E, Pérez-Zepeda MU, Crabtree DR, Nicholson C, et al. Recent developments in frailty identification, management, risk factors and prevention: A narrative review of leading journals in geriatrics and gerontology. Ageing Res Rev. 2023;91:102082. 37797723 10.1016/j.arr.2023.102082

[5] Hirose T, Sawaya Y, Ishizaka M, Hashimoto N, Kubo A, Urano T. Frailty under COVID-19 pandemic in Japan: changes in prevalence of frailty from 2017 to 2021. J Am Geriatr Soc. 2023;71(5):1603–9. 36647923 10.1111/jgs.18237

[6] Yamada M, Arai H. Does the COVID-19 pandemic robustly influence the incidence of frailty? Geriatr Gerontol Int. 2021;21(8):754–5. 34216187 10.1111/ggi.14233 PMC8444923

[7] Pilotto A, Custodero C, Zora S, Poli S, Senesi B, Prete C, Tavella E, Veronese N, Zini E, Torrigiani C, et al. Frailty trajectories in community-dwelling older adults during COVID-19 pandemic: the PRESTIGE study. Eur J Clin Invest. 2022;52(12):e13838. 35842830 10.1111/eci.13838 PMC9350279

[8] Pizano-Escalante MG, Anaya-Esparza LM, Nuño K, Rodríguez-Romero JJ, Gonzalez-Torres S, López-de la Mora DA, Villagrán Z. Direct and indirect effects of COVID-19 in frail elderly: interventions and recommendations. J Pers Med 2021, 11(10). 10.3390/jpm11100999 PMC8539433 34683141

[9] Holland C, Garner I, Simpson J, Eccles F, Pardo EN, Marr C, Varey S. Impacts of COVID-19 lockdowns on frailty and wellbeing in older people and those living with long-term conditions. Adv Clin Exp Med. 2021;30(11):1111–4. 34821484 10.17219/acem/144135

[10] Padilha de Lima A, Macedo Rogero M, Araujo Viel T, Garay-Malpartida HM, Aprahamian I, Lima Ribeiro SM. Interplay between inflammaging, frailty and nutrition in Covid-19: preventive and adjuvant treatment perspectives. J Nutr Health Aging. 2022;26(1):67–76. 35067706 10.1007/s12603-021-1720-5 PMC8713542

[11] Ammar A, Brach M, Trabelsi K, Chtourou H, Boukhris O, Masmoudi L, Bouaziz B, Bentlage E, How D, Ahmed M et al. Effects of COVID-19 home confinement on eating behaviour and physical activity: results of the ECLB-COVID19 international online survey. Nutrients 2020, 12(6). 10.3390/nu12061583 PMC7352706 32481594

[12] Zhu A, Yan L, Wu C, Ji JS. Residential greenness and frailty among older adults: A longitudinal cohort in China. J Am Med Dir Assoc. 2020;21(6):759–e765752. 31870716 10.1016/j.jamda.2019.11.006 PMC7303951

[13] Di Bari M, Tonarelli F, Balzi D, Giordano A, Ungar A, Baldasseroni S, Onder G, Mechi MT, Carreras G. COVID-19, vulnerability, and Long-Term mortality in hospitalized and nonhospitalized older persons. J Am Med Dir Assoc. 2022;23(3):414–e420411. 34990587 10.1016/j.jamda.2021.12.009 PMC8673732

[14] Si H, Jin Y, Qiao X, Tian X, Liu X, Wang C. Predictive performance of 7 frailty instruments for short-term disability, falls and hospitalization among Chinese community-dwelling older adults: A prospective cohort study. Int J Nurs Stud. 2021;117:103875. 33621721 10.1016/j.ijnurstu.2021.103875

[15] Kojima G, Iliffe S, Walters K. Frailty index as a predictor of mortality: a systematic review and meta-analysis. Age Ageing. 2018;47(2):193–200. 29040347 10.1093/ageing/afx162

[16] Zhou Q, Li Y, Gao Q, Yuan H, Sun L, Xi H, Wu W. Prevalence of frailty among Chinese Community-Dwelling older adults: A systematic review and Meta-Analysis. Int J Public Health. 2023;68:1605964. 37588041 10.3389/ijph.2023.1605964 PMC10425593

[17] Dent E, Martin FC, Bergman H, Woo J, Romero-Ortuno R, Walston JD. Management of frailty: opportunities, challenges, and future directions. Lancet. 2019;394(10206):1376–86. 31609229 10.1016/S0140-6736(19)31785-4

[18] Albert SM. The dynamics of frailty among older adults. JAMA Netw Open. 2019;2(8):e198438. 31373644 10.1001/jamanetworkopen.2019.8438

[19] Hoogendijk EO, Dent E. Trajectories, transitions, and trends in frailty among older adults: A review. Ann Geriatr Med Res. 2022;26(4):289–95. 36503183 10.4235/agmr.22.0148 PMC9830071

[20] Bray NW, Smart RR, Jakobi JM, Jones GR. Exercise prescription to reverse frailty. Appl Physiol Nutr Metab. 2016;41(10):1112–6. 27649859 10.1139/apnm-2016-0226

[21] Lavie CJ, Arena R, Swift DL, Johannsen NM, Sui X, Lee DC, Earnest CP, Church TS, O’Keefe JH, Milani RV, et al. Exercise and the cardiovascular system: clinical science and cardiovascular outcomes. Circ Res. 2015;117(2):207–19. 26139859 10.1161/CIRCRESAHA.117.305205 PMC4493772

[22] Powers SK, Jackson MJ. Exercise-induced oxidative stress: cellular mechanisms and impact on muscle force production. Physiol Rev. 2008;88(4):1243–76. 18923182 10.1152/physrev.00031.2007 PMC2909187

[23] Nowacka-Chmielewska M, Grabowska K, Grabowski M, Meybohm P, Burek M, Małecki A. Running from stress: Neurobiological mechanisms of Exercise-Induced stress resilience. Int J Mol Sci 2022, 23(21). 10.3390/ijms232113348 PMC9654650 36362131

[24] Bull FC, Al-Ansari SS, Biddle S, Borodulin K, Buman MP, Cardon G, Carty C, Chaput JP, Chastin S, Chou R, et al. World health organization 2020 guidelines on physical activity and sedentary behaviour. Br J Sports Med. 2020;54(24):1451–62. 33239350 10.1136/bjsports-2020-102955 PMC7719906

[25] Schoenfeld BJ, Ogborn D, Krieger JW. Dose-response relationship between weekly resistance training volume and increases in muscle mass: A systematic review and meta-analysis. J Sports Sci. 2017;35(11):1073–82. 27433992 10.1080/02640414.2016.1210197

[26] Taylor JA, Greenhaff PL, Bartlett DB, Jackson TA, Duggal NA, Lord JM. Multisystem physiological perspective of human frailty and its modulation by physical activity. Physiol Rev. 2023;103(2):1137–91. 36239451 10.1152/physrev.00037.2021 PMC9886361

[27] Zhang YJ, Yao Y, Zhang PD, Li ZH, Zhang P, Li FR, Wang ZH, Liu D, Lv YB, Kang L, et al. Association of regular aerobic exercises and neuromuscular junction variants with incidence of frailty: an analysis of the Chinese longitudinal health and longevity survey. J Cachexia Sarcopenia Muscle. 2021;12(2):350–7. 33527771 10.1002/jcsm.12658 PMC8061381

[28] da Silva VD, Tribess S, Meneguci J, Sasaki JE, Garcia-Meneguci CA, Carneiro JAO, Virtuoso JS Jr. Association between frailty and the combination of physical activity level and sedentary behavior in older adults. BMC Public Health. 2019;19(1):709. 31174515 10.1186/s12889-019-7062-0 PMC6555975

[29] Rogers NT, Marshall A, Roberts CH, Demakakos P, Steptoe A, Scholes S. Physical activity and trajectories of frailty among older adults: evidence from the english longitudinal study of ageing. PLoS ONE. 2017;12(2):e0170878. 28152084 10.1371/journal.pone.0170878 PMC5289530

[30] Wei M, He S, Meng D, Yang G, Wang Z. Hybrid exercise program enhances physical fitness and reverses frailty in older adults: insights and predictions from machine learning. J Nutr Health Aging. 2023;27(10):894–902.37960913 10.1007/s12603-023-1991-0

[31] Huang CY, Mayer PK, Wu MY, Liu DH, Wu PC, Yen HR. The effect of Tai Chi in elderly individuals with sarcopenia and frailty: A systematic review and meta-analysis of randomized controlled trials. Ageing Res Rev. 2022;82:101747. 36223875 10.1016/j.arr.2022.101747

[32] Zhang X, Tan SS, Franse CB, Bilajac L, Alhambra-Borrás T, Garcés-Ferrer J, Verma A, Williams G, Clough G, Koppelaar E, et al. Longitudinal association between physical activity and frailty among Community-Dwelling older adults. J Am Geriatr Soc. 2020;68(7):1484–93. 32196638 10.1111/jgs.16391 PMC7383618

[33] Lee SY, Nyunt MSZ, Gao Q, Gwee X, Chua DQL, Yap KB, Wee SL, Ng TP. Longitudinal associations of housework with frailty and mortality in older adults: Singapore longitudinal ageing study 2. BMC Geriatr. 2022;22(1):962. 36514054 10.1186/s12877-022-03591-6 PMC9749321

[34] Tison GH, Avram R, Kuhar P, Abreau S, Marcus GM, Pletcher MJ, Olgin JE. Worldwide effect of COVID-19 on physical activity: A descriptive study. Ann Intern Med. 2020;173(9):767–70. 32598162 10.7326/M20-2665 PMC7384265

[35] Steptoe A, Breeze E, Banks J, Nazroo J. Cohort profile: the english longitudinal study of ageing. Int J Epidemiol. 2013;42(6):1640–8. 23143611 10.1093/ije/dys168 PMC3900867

[36] Ray J, Popli G, Fell G. Association of cognition and Age-Related hearing impairment in the english longitudinal study of ageing. JAMA Otolaryngol Head Neck Surg. 2018;144(10):876–82. 30193368 10.1001/jamaoto.2018.1656 PMC6233824

[37] Hamilton OS, Steptoe A, Ajnakina O. Polygenic predisposition, sleep duration, and depression: evidence from a prospective population-based cohort. Transl Psychiatry. 2023;13(1):323. 37857612 10.1038/s41398-023-02622-z PMC10587060

[38] Sun J, Lin J, Shen W, Ding P, Yang W, Huang L, Chen H. Associations of body mass index, waist circumference and the weight-adjusted waist index with daily living ability impairment in older Chinese people: A cross-sectional study of the Chinese longitudinal healthy longevity survey. Diabetes Obes Metabolism. 2024;26(9):4069–77. 10.1111/dom.15762 38962934

[39] Liang Z, Jin W, Huang L, Chen H. Body mass index, waist circumference, hip circumference, abdominal volume index, and cognitive function in older Chinese people: a nationwide study. BMC Geriatr. 2024;24(1):925. 39516791 10.1186/s12877-024-05521-0 PMC11546056

[40] Mitnitski AB, Mogilner AJ, Rockwood K. Accumulation of deficits as a proxy measure of aging. ScientificWorldJournal. 2001;1:323–36. 12806071 10.1100/tsw.2001.58 PMC6084020

[41] Searle SD, Mitnitski A, Gahbauer EA, Gill TM, Rockwood K. A standard procedure for creating a frailty index. BMC Geriatr. 2008;8:24. 18826625 10.1186/1471-2318-8-24 PMC2573877

[42] He D, Qiu Y, Yan M, Zhou T, Cheng Z, Li J, Wu Q, Liu Z, Zhu Y. Associations of metabolic heterogeneity of obesity with frailty progression: results from two prospective cohorts. J Cachexia Sarcopenia Muscle. 2023;14(1):632–41. 36575595 10.1002/jcsm.13169 PMC9891922

[43] Mindell J, Biddulph JP, Hirani V, Stamatakis E, Craig R, Nunn S, Shelton N. Cohort profile: the health survey for England. Int J Epidemiol. 2012;41(6):1585–93. 22253315 10.1093/ije/dyr199

[44] Pedisic Z, Shrestha N, Kovalchik S, Stamatakis E, Liangruenrom N, Grgic J, Titze S, Biddle SJ, Bauman AE, Oja P. Is running associated with a lower risk of all-cause, cardiovascular and cancer mortality, and is the more the better? A systematic review and meta-analysis. Br J Sports Med. 2020;54(15):898–905. 31685526 10.1136/bjsports-2018-100493

[45] O’Donovan G, Petermann-Rocha F, Ferrari G, Medina C, Ochoa-Rosales C, Sarmiento OLL, Ibáñez A. Associations of the ‘weekend warrior’ physical activity pattern with mild dementia: findings from the Mexico City Prospective Study. Br J Sports Med. 2024;59(5):325–332. 10.1136/bjsports-2024-108460 39472031

[46] Alexandre TDS, Scholes S, Santos JLF, de Oliveira C. Dynapenic abdominal obesity as a risk factor for worse trajectories of ADL disability among older adults: the ELSA cohort study. J Gerontol Biol Sci Med Sci. 2019;74(7):1112–8. 10.1093/gerona/gly182 PMC6580691 30165562

[47] Luiz MM, Máximo R, Oliveira DC, Ramírez PC, de Souza AF, Delinocente MLB, Steptoe A, de Oliveira C, Alexandre T. Association of serum 25-Hydroxyvitamin D deficiency with risk of incidence of disability in basic activities of daily living in adults > 50 years of age. J Nutr. 2020;150(11):2977–84. 32937653 10.1093/jn/nxaa258 PMC7675030

[48] Demakakos P, Hamer M, Stamatakis E, Steptoe A. Low-intensity physical activity is associated with reduced risk of incident type 2 diabetes in older adults: evidence from the english longitudinal study of ageing. Diabetologia. 2010;53(9):1877–85. 20495973 10.1007/s00125-010-1785-x

[49] Hamer M, de Oliveira C, Demakakos P. Non-exercise physical activity and survival: english longitudinal study of ageing. Am J Prev Med. 2014;47(4):452–60. 25049216 10.1016/j.amepre.2014.05.044

[50] Dhalwani NN, O’Donovan G, Zaccardi F, Hamer M, Yates T, Davies M, Khunti K. Long terms trends of Multimorbidity and association with physical activity in older english population. Int J Behav Nutr Phys Act. 2016;13:8. 26785753 10.1186/s12966-016-0330-9 PMC4717631

[51] Wanigatunga AA, Cai Y, Urbanek JK, Mitchell CM, Roth DL, Miller ER, Michos ED, Juraschek SP, Walston J, Xue QL, et al. Objectively measured patterns of daily physical activity and phenotypic frailty. J Gerontol Biol Sci Med Sci. 2022;77(9):1882–9. 10.1093/gerona/glab278 PMC9434427 34562073

[52] Kikuchi H, Inoue S, Amagasa S, Fukushima N, Machida M, Murayama H, Fujiwara T, Chastin S, Owen N, Shobugawa Y. Associations of older adults’ physical activity and bout-specific sedentary time with frailty status: compositional analyses from the NEIGE study. Exp Gerontol. 2021;143:111149. 33181316 10.1016/j.exger.2020.111149

[53] Lorenzo-López L, Maseda A, de Labra C, Regueiro-Folgueira L, Rodríguez-Villamil JL, Millán-Calenti JC. Nutritional determinants of frailty in older adults: A systematic review. BMC Geriatr. 2017;17(1):108. 28506216 10.1186/s12877-017-0496-2 PMC5433026

[54] Kojima G, Taniguchi Y, Iliffe S, Jivraj S, Walters K. Transitions between frailty States among community-dwelling older people: A systematic review and meta-analysis. Ageing Res Rev. 2019;50:81–8. 30659942 10.1016/j.arr.2019.01.010

[55] Zhu Y, Fan J, Lv J, Guo Y, Pei P, Yang L, Chen Y, Du H, Li F, Yang X, et al. Maintaining healthy sleep patterns and frailty transitions: a prospective Chinese study. BMC Med. 2022;20(1):354. 36266610 10.1186/s12916-022-02557-0 PMC9585775

[56] Zhang P, Zhong J, Liu X, Sun W. The association between dynamic changes in vitamin D and frailty alterations: A prospective analysis of UK biobank participants. J Cachexia Sarcopenia Muscle. 2024;15(5):1722–32. 38923848 10.1002/jcsm.13525 PMC11446684

[57] Qiu Y, Fernández-García B, Lehmann HI, Li G, Kroemer G, López-Otín C, Xiao J. Exercise sustains the hallmarks of health. J Sport Health Sci. 2023;12(1):8–35. 36374766 10.1016/j.jshs.2022.10.003 PMC9923435

[58] Naylor E, Penev PD, Orbeta L, Janssen I, Ortiz R, Colecchia EF, Keng M, Finkel S, Zee PC. Daily social and physical activity increases slow-wave sleep and daytime neuropsychological performance in the elderly. Sleep. 2000;23(1):87–95. 10678469

[59] Manfredini F, Mallamaci F, D’Arrigo G, Baggetta R, Bolignano D, Torino C, Lamberti N, Bertoli S, Ciurlino D, Rocca-Rey L, et al. Exercise in patients on dialysis: A multicenter, randomized clinical trial. J Am Soc Nephrol. 2017;28(4):1259–68. 27909047 10.1681/ASN.2016030378 PMC5373448

[60] Lai X, Zhu H, Wu Z, Chen B, Jiang Q, Du H, Huo X. Dose-response effects of resistance training on physical function in frail older Chinese adults: A randomized controlled trial. J Cachexia Sarcopenia Muscle. 2023;14(6):2824–34. 37875291 10.1002/jcsm.13359 PMC10751415

[61] Fiatarone MA, O’Neill EF, Ryan ND, Clements KM, Solares GR, Nelson ME, Roberts SB, Kehayias JJ, Lipsitz LA, Evans WJ. Exercise training and nutritional supplementation for physical frailty in very elderly people. N Engl J Med. 1994;330(25):1769–75. 8190152 10.1056/NEJM199406233302501

[62] Cunningham C, R OS, Caserotti P, Tully MA. Consequences of physical inactivity in older adults: A systematic review of reviews and meta-analyses. Scand J Med Sci Sports. 2020;30(5):816–27. 32020713 10.1111/sms.13616

[63] Figueiredo VC, de Salles BF, Trajano GS. Volume for muscle hypertrophy and health outcomes: the most effective variable in resistance training. Sports Med. 2018;48(3):499–505. 29022275 10.1007/s40279-017-0793-0

