# Supplementary material for: Association between physical activity and frailty transitions in middle-aged and older adults: a nationwide longitudinal study
Source: Int J Behav Nutr Phys Act. 2025 Mar 10;22:31. doi: 10.1186/s12966-025-01725-8 (PMC11895274; doi:10.1186/s12966-025-01725-8)
Supplement: Supplementary file 1 — Supplementary Material 1 [file 12966_2025_1725_MOESM1_ESM.docx]

**Supplementary Table S1** The 32 items used to construct the frailty index

| No | Description of the items | Cut-off value |
| --- | --- | --- |
| 1 | Self-reported physician diagnosed hypertension | Yes = 1, No = 0 |
| 2 | Self-reported physician diagnosed diabetes | Yes = 1, No = 0 |
| 3 | Self-reported physician diagnosed heart disease | Yes = 1, No = 0 |
| 4 | Self-reported physician diagnosed stroke | Yes = 1, No = 0 |
| 5 | Self-reported physician diagnosed cancer | Yes = 1, No = 0 |
| 6 | Self-reported physician diagnosed arthritis | Yes = 1, No = 0 |
| 7 | Self-reported physician diagnosed chronic lung disease | Yes = 1, No = 0 |
| 8 | Self-reported physician diagnosed asthma | Yes = 1, No = 0 |
| 9 | Self-reported physician diagnosed any emotional, nervous, or psychiatric problems | Yes = 1, No = 0 |
| 10 | Self-reported physician diagnosed memory-related disease | Yes = 1, No = 0 |
| 11 | Self-reported eyesight (while using lenses if appropriate) | Poor or fair = 1, excellent, very good, or good = 0 |
| 12 | Self-reported hearing (while using hearing aid if appropriate) | Poor or fair = 1, excellent, very good, or good = 0 |
| 13 | Self-reported general health status | Poor or fair = 1, excellent, very good, or good = 0 |
| 14 | Difficulty with dressing | Yes = 1, No = 0 |
| 15 | Difficulty with bathing or showering | Yes = 1, No = 0 |
| 16 | Difficulty with eating | Yes = 1, No = 0 |
| 17 | Difficulty with getting in and out of bed | Yes = 1, No = 0 |
| 18 | Difficulty with using the toilet | Yes = 1, No = 0 |
| 19 | Difficulty with managing money | Yes = 1, No = 0 |
| 20 | Difficulty with taking medications | Yes = 1, No = 0 |
| 21 | Difficulty with shopping for groceries | Yes = 1, No = 0 |
| 22 | Difficulty with preparing meals | Yes = 1, No = 0 |
| 23 | Difficulty with doing housework | Yes = 1, No = 0 |
| 24 | Mobility: difficulty with walking 100 yards | Yes = 1, No = 0 |
| 25 | Mobility: difficulty with getting up from a chair after sitting for long periods | Yes = 1, No = 0 |
| 26 | Mobility: difficulty with climbing several flights of stairs without resting | Yes = 1, No = 0 |
| 27 | Mobility: difficulty with lifting or carrying weights over 10 pounds | Yes = 1, No = 0 |
| 28 | Mobility: difficulty with picking up a coin from the table | Yes = 1, No = 0 |
| 29 | Mobility: difficulty with stooping, kneeling, or crouching | Yes = 1, No = 0 |
| 30 | Mobility: difficulty with reaching arms above shoulder level | Yes = 1, No = 0 |
| 31 | Depression: CESD-8 questionnaire | CESD-8 ≥ 4 =1, <4 =0 |
| 32 | Cognition: (memory test score + orientation test score) **/** 14 | Continuous, ranging from 0 to 1 |

Heart disease indicates the angina, myocardial infarction, congestive heart failure, heart murmur, arrhythmia, or other heart problems.

Memory-related disease indicates Alzheimer’s disease or dementia, Parkinson's disease, or other serious memory impairment.

Depression is evaluated using Center for Epidemiologic Studies Depression Scale (CESD). CESD-8 is used, and the total score ranges from 0 to 8. The higher score indicates more severe depressive symptoms.

The memory score is the average of words that are not recalled in the immediate and delayed word recall tasks. The memory score ranges from 0 to 10. The orientation test comprises four questions about the day of the week, the month, the date of the month, and the year. One point is given for each wrong answer, and the range is from 0 to 4.

**Supplementary Table S2** Coronavirus symptoms.

| **Variables** | **Transition from non-frailty to frailty**  **(n = 4087)** | **Transition from frailty to non-frailty**  **(n = 509)** |
| --- | --- | --- |
| High temperature, n (%) |  |  |
| No | 3931 (96.2) | 485 (95.3) |
| Yes | 156 (3.8) | 24 (4.7) |
| A new continuous cough, n (%) |  |  |
| No | 3938 (96.4) | 480 (94.3) |
| Yes | 149 (3.6) | 29 (5.7) |
| Shortness of breath or trouble breathing, n (%) |  |  |
| No | 3896 (95.3) | 430 (84.5) |
| Yes | 191 (4.7) | 79 (15.5) |
| Fatigue, n (%) |  |  |
| No | 3573 (87.4) | 381 (74.9) |
| Yes | 514 (12.6) | 128 (25.1) |
| Loss of sense of smell or taste, n (%) |  |  |
| No | 3946 (96.6) | 491 (96.5) |
| Yes | 141 (3.4) | 18 (3.5) |
| Diarrhoea, n (%) |  |  |
| No | 3887 (95.1) | 469 (92.1) |
| Yes | 200 (4.9) | 40 (7.9) |
| Abdominal pain, n (%) |  |  |
| No | 3923 (96.0) | 465 (91.4) |
| Yes | 164 (4.0) | 44 (8.6) |
| Loss of appetite, n (%) |  |  |
| No | 3941 (96.4) | 465 (91.4) |
| Yes | 146 (3.6) | 44 (8.6) |
| Number of coronavirus symptoms, n (%) |  |  |
| 0 | 3288 (80.5) | 331 (65.0) |
| 1 | 366 (9.0) | 67 (13.2) |
| 2 | 216 (5.3) | 54 (10.6) |
| 3 | 94 (2.3) | 27 (5.3) |
| 4 | 68 (1.7) | 11 (2.2) |
| 5 | 34 (0.8) | 10 (2.0) |
| 6 | 10 (0.2) | 7 (1.4) |
| 7 | 9 (0.2) | 2 (0.4) |
| 8 | 2 (0.0) | 0 (0.0) |

**Supplementary Table S3** Association of the comprehensive PA level with frailty worsening.

| **Variables** | **Model 1** | | **Model 2** | |
| --- | --- | --- | --- | --- |
|  | **OR (95% CI)** | ***P*** | **OR (95% CI)** | ***P*** |
| **PA levels** |  |  |  |  |
| Inactive | 1 (Reference) |  | 1 (Reference) |  |
| Low | 0.68 (0.43-1.10) | .117 | 0.70 (0.42-1.15) | .159 |
| Moderate | 0.27 (0.17-0.41) | **<.001** | 0.32 (0.20-0.51) | **<.001** |
| High | 0.12 (0.07-0.19) | **<.001** | 0.19 (0.11-0.31) | **<.001** |

Model 1: Unadjusted.

Model 2: Adjusted for age, sex, ethnicity, education level, marital status, smoking status, drinker status, use of high blood pressure medication and use of diabetes medication.

PA, Physical activity.

**Supplementary Table S4** Association of the comprehensive PA level with frailty improvement.

| **Variables** | **Model 1** | | **Model 2** | |
| --- | --- | --- | --- | --- |
|  | **OR (95% CI)** | ***P*** | **OR (95% CI)** | ***P*** |
| **PA levels** |  |  |  |  |
| Inactive | 1 (Reference) |  | 1 (Reference) |  |
| Low | 1.79 (0.91-3.51) | .092 | 2.06 (1.02-4.15) | **.044** |
| Moderate | 2.98 (1.53-5.80) | **.001** | 3.30 (1.65-6.63) | **<.001** |
| High | 2.59 (0.98-6.86) | .055 | 2.69 (0.98-7.38) | .054 |

Model 1: Unadjusted.

Model 2: Adjusted for age, sex, ethnicity, education level, marital status, smoking status, drinker status, use of high blood pressure medication and use of diabetes medication.

PA, Physical activity.

**Supplementary Table S5** Subgroup analysis.

| **Variables** | **OR (95% CI)** | | | | ***P* for interaction** |
| --- | --- | --- | --- | --- | --- |
|  | **Inactive** | **Low** | **Moderate** | **High** |  |
| **Transition from non-frailty to frailty** | | | | | |
| **Age** |  |  |  |  | .979 |
| 50-64 | 1 (Reference) | 0.68 (0.24-1.94) | 0.27 (0.10-0.70) | 0.19 (0.07-0.50) |  |
| >=65 | 1 (Reference) | 0.71 (0.40-1.26) | 0.31 (0.19-0.52) | 0.16 (0.09-0.29) |  |
| **Sex** |  |  |  |  | .592 |
| Male | 1 (Reference) | 0.49 (0.23-1.02) | 0.25 (0.13-0.46) | 0.15 (0.08-0.29) |  |
| Female | 1 (Reference) | 0.99 (0.48-2.06) | 0.40 (0.20-0.80) | 0.21 (0.10-0.43) |  |
| **Transition from frailty to non-frailty** | | | | | |
| **Age** |  |  |  |  | .449 |
| 50-64 | 1 (Reference) | 1.06 (0.29-3.85) | 1.74 (0.53-5.71) | 0.82 (0.07-9.30) |  |
| >=65 | 1 (Reference) | 2.82 (1.16-6.88) | 4.83 (1.95-11.93) | 4.25 (1.29-14.03) |  |
| **Sex** |  |  |  |  | .678 |
| Male | 1 (Reference) | 1.85 (0.65-5.23) | 2.24 (0.80-6.29) | 2.13 (0.43-10.59) |  |
| Female | 1 (Reference) | 2.09 (0.74-5.92) | 3.95 (1.40-11.18) | 3.36 (0.82-13.75) |  |

All analyses were adjusted for age, sex, ethnicity, education level, marital status, smoking status, drinker status, use of high blood pressure medication and use of diabetes medication.

PA, Physical activity.

**Supplementary Table S6** Association of the comprehensive PA level with frailty worsening (n = 4087).

| **Variables** | **Model 1** | | **Model 2** | |
| --- | --- | --- | --- | --- |
|  | **OR (95% CI)** | ***P*** | **OR (95% CI)** | ***P*** |
| **PA levels** |  |  |  |  |
| Inactive | 1 (Reference) |  | 1 (Reference) |  |
| Low | 0.79 (0.47-1.33) | .379 | 0.80 (0.46-1.38) | .415 |
| Moderate | 0.30 (0.19-0.48) | **<.001** | 0.35 (0.21-0.58) | **<.001** |
| High | 0.14 (0.08-0.23) | **<.001** | 0.21 (0.12-0.36) | **<.001** |

Model 1: Unadjusted.

Model 2: Adjusted for age, sex, ethnicity, education level, marital status, smoking status, drinker status, use of high blood pressure medication, use of diabetes medication, and number of coronavirus symptoms.

PA, Physical activity.

**Supplementary Table S7** Association of the comprehensive PA level with frailty improvement (n = 509).

| **Variables** | **Model 1** | | **Model 2** | |
| --- | --- | --- | --- | --- |
|  | **OR (95% CI)** | ***P*** | **OR (95% CI)** | ***P*** |
| **PA levels** |  |  |  |  |
| Inactive | 1 (Reference) |  | 1 (Reference) |  |
| Low | 1.81 (0.89-3.65) | .100 | 2.18 (1.03-4.59) | **.040** |
| Moderate | 2.95 (1.48-5.89) | **.002** | 3.66 (1.74-7.68) | **<.001** |
| High | 2.20 (0.79-6.14) | .130 | 2.34 (0.79-6.88) | .124 |

Model 1: Unadjusted.

Model 2: Adjusted for age, sex, ethnicity, education level, marital status, smoking status, drinker status, use of high blood pressure medication, use of diabetes medication, and number of coronavirus symptoms.

PA, Physical activity.

**Supplementary Table S8** Subgroup analysis.

| **Variables** | **OR (95% CI)** | | | | ***P* for interaction** |
| --- | --- | --- | --- | --- | --- |
|  | **Inactive** | **Low** | **Moderate** | **High** |  |
| **Transition from non-frailty to frailty** | | | | | |
| **Age** |  |  |  |  | .908 |
| 50-64 | 1 (Reference) | 0.77 (0.25-2.34) | 0.25 (0.09-0.71) | 0.17 (0.06-0.50) |  |
| >=65 | 1 (Reference) | 0.85 (0.45-1.59) | 0.37 (0.21-0.65) | 0.20 (0.11-0.37) |  |
| **Sex** |  |  |  |  | .618 |
| Male | 1 (Reference) | 0.60 (0.27-1.37) | 0.27 (0.13-0.54) | 0.18 (0.09-0.37) |  |
| Female | 1 (Reference) | 1.11 (0.51-2.45) | 0.45 (0.21-0.94) | 0.22 (0.10-0.49) |  |
| **Transition from frailty to non-frailty** | | | | | |
| **Age** |  |  |  |  | .236 |
| 50-64 | 1 (Reference) | 0.99 (0.26-3.79) | 1.51 (0.43-5.26) | 0.42 (0.03-5.11) |  |
| >=65 | 1 (Reference) | 3.38 (1.27-8.96) | 6.41 (2.37-17.34) | 4.73 (1.27-17.65) |  |
| **Sex** |  |  |  |  | .921 |
| Male | 1 (Reference) | 2.02 (0.65-6.25) | 2.99 (0.97-9.17) | 2.21 (0.41-11.87) |  |
| Female | 1 (Reference) | 2.19 (0.75-6.39) | 4.10 (1.39-12.12) | 2.75 (0.60-12.55) |  |

All analyses were adjusted for age, sex, ethnicity, education level, marital status, smoking status, drinker status, use of high blood pressure medication, use of diabetes medication, and number of coronavirus symptoms.

PA, Physical activity.

**Supplementary Table S9** Association between PA frequency and frailty worsening (n = 4087).

| **Variables** | **Model 1** | | **Model 2** | |
| --- | --- | --- | --- | --- |
|  | **OR (95% CI)** | ***P*** | **OR (95% CI)** | ***P*** |
| **Vigorous PA** |  |  |  |  |
| <1 per week | 1 (Reference) |  | 1 (Reference) |  |
| ≥1 per week | 0.36 (0.27-0.47) | **<.001** | 0.49 (0.36-0.65) | **<.001** |
| **Moderate PA** |  |  |  |  |
| <1 per week | 1 (Reference) |  | 1 (Reference) |  |
| ≥1 per week | 0.28 (0.22-0.37) | **<.001** | 0.37 (0.28-0.49) | **<.001** |
| **Mild PA** |  |  |  |  |
| <1 per week | 1 (Reference) |  | 1 (Reference) |  |
| ≥1 per week | 0.38 (0.26-0.55) | **<.001** | 0.41 (0.27-0.61) | **<.001** |
| **PA (Any)** |  |  |  |  |
| <1 per week | 1 (Reference) |  | 1 (Reference) |  |
| ≥1 per week | 0.27 (0.17-0.43) | **<.001** | 0.35 (0.21-0.58) | **<.001** |

Model 1: Unadjusted.

Model 2: Adjusted for age, sex, ethnicity, education level, marital status, smoking status, drinker status, use of high blood pressure medication, use of diabetes medication, and number of coronavirus symptoms.

PA, Physical activity.

**Supplementary Table S10** Association between PA frequency and frailty improvement (n = 509).

| **Variables** | **Model 1** | | **Model 2** | |
| --- | --- | --- | --- | --- |
|  | **OR (95% CI)** | ***P*** | **OR (95% CI)** | ***P*** |
| **Vigorous PA** |  |  |  |  |
| <1 per week | 1 (Reference) |  | 1 (Reference) |  |
| ≥1 per week | 1.12 (0.47-2.66) | .790 | 1.01 (0.41-2.49) | .988 |
| **Moderate PA** |  |  |  |  |
| <1 per week | 1 (Reference) |  | 1 (Reference) |  |
| ≥1 per week | 1.95 (1.24-3.06) | **.004** | 2.10 (1.30-3.39) | **.002** |
| **Mild PA** |  |  |  |  |
| <1 per week | 1 (Reference) |  | 1 (Reference) |  |
| ≥1 per week | 2.51 (1.32-4.78) | **.005** | 3.17 (1.60-6.31) | **.001** |
| **PA (Any)** |  |  |  |  |
| <1 per week | 1 (Reference) |  | 1 (Reference) |  |
| ≥1 per week | 2.30 (1.20-4.38) | **.011** | 2.75 (1.38-5.47) | **.004** |

Model 1: Unadjusted.

Model 2: Adjusted for age, sex, ethnicity, education level, marital status, smoking status, drinker status, use of high blood pressure medication, use of diabetes medication, and number of coronavirus symptoms.

PA, Physical activity.

**Supplementary Table S11** Association between PA changes and frailty worsening (n = 4086).

| **Variables** | ***n*** | **Model 1** | | **Model 2** | |
| --- | --- | --- | --- | --- | --- |
|  |  | **OR (95% CI)** | ***P*** | **OR (95% CI)** | ***P*** |
| **Vigorous PA** |  |  |  |  |  |
| Inactive | 2040 | 1 (Reference) |  | 1 (Reference) |  |
| Inactive to Active | 385 | 0.30 (0.18-0.52) | **<.001** | 0.39 (0.23-0.67) | **<.001** |
| Active to Inactive | 711 | 0.57 (0.41-0.78) | **<.001** | 0.68 (0.49-0.94) | **.021** |
| Consistently active | 950 | 0.14 (0.08-0.22) | **<.001** | 0.21 (0.13-0.35) | **<.001** |
| **Moderate PA** |  |  |  |  |  |
| Inactive | 201 | 1 (Reference) |  | 1 (Reference) |  |
| Inactive to Active | 293 | 0.34 (0.21-0.54) | **<.001** | 0.40 (0.25-0.65) | **<.001** |
| Active to Inactive | 510 | 0.62 (0.43-0.90) | **.013** | 0.69 (0.47-1.02) | .064 |
| Consistently active | 3082 | 0.10 (0.07-0.14) | **<.001** | 0.14 (0.09-0.20) | **<.001** |
| **Mild PA** |  |  |  |  |  |
| Inactive | 53 | 1 (Reference) |  | 1 (Reference) |  |
| Inactive to Active | 159 | 0.41 (0.19-0.86) | **.018** | 0.47 (0.21-1.05) | .066 |
| Active to Inactive | 351 | 0.58 (0.30-1.11) | .099 | 0.57 (0.28-1.15) | .118 |
| Consistently active | 3523 | 0.17 (0.09-0.31) | **<.001** | 0.20 (0.10-0.39) | **<.001** |

Model 1: Unadjusted.

Model 2: Adjusted for age, sex, ethnicity, education level, marital status, smoking status, drinker status, use of high blood pressure medication, use of diabetes medication, and number of coronavirus symptoms.

PA, Physical activity.

**Supplementary Table S12** Association between PA changes and frailty improvement (n = 509).

| **Variables** | ***n*** | **Model 1** | | **Model 2** | |
| --- | --- | --- | --- | --- | --- |
|  |  | **OR (95% CI)** | ***P*** | **OR (95% CI)** | ***P*** |
| **Moderate PA** |  |  |  |  |  |
| Inactive | 244 | 1 (Reference) |  | 1 (Reference) |  |
| Inactive to Active | 77 | 1.95 (1.01-3.76) | **.047** | 1.94 (0.96-3.91) | .064 |
| Active to Inactive | 80 | 1.46 (0.73-2.90) | .284 | 1.72 (0.84-3.55) | .139 |
| Consistently active | 108 | 3.16 (1.81-5.49) | **<.001** | 3.26 (1.79-5.94) | **<.001** |
| **Mild PA** |  |  |  |  |  |
| Inactive | 82 | 1 (Reference) |  | 1 (Reference) |  |
| Inactive to Active | 39 | 2.30 (0.69-7.67) | .174 | 2.39 (0.68-8.42) | .175 |
| Active to Inactive | 100 | 2.59 (0.97-6.92) | .057 | 3.40 (1.22-9.47) | **.019** |
| Consistently active | 288 | 3.84 (1.60-9.21) | **.003** | 4.89 (1.95-12.25) | **<.001** |

Model 1: Unadjusted.

Model 2: Adjusted for age, sex, ethnicity, education level, marital status, smoking status, drinker status, use of high blood pressure medication, use of diabetes medication, and number of coronavirus symptoms.

PA, Physical activity.
